# The role of chronic disorders in psychotherapy

**DOI:** 10.1192/j.eurpsy.2022.866

**Published:** 2022-09-01

**Authors:** M. Linden

**Affiliations:** Charité University Medicine Berlin, Psychosomatic Medicine And Rehabilitation, Berlin, Germany

**Keywords:** chronic disorder, Psychotherapy, impairment

## Abstract

**Introduction:**

Many mental disorders take a chronic course, associated with disability and/or participation restrictions. This is well recognized in social psychiatry. It is assumed that in psychotherapy milder disorders are seen, but there are no data available in this regard. In a survey in outpatient psychotherapy the rate of patients with chronic disorders and associated impairment was assessed.

**Objectives:**

Goal of the present study was to assess the prevalence and meaning of long term and prevailing disorders in psychotherapy

**Methods:**

A total of 131 psychotherapists (43.5% psychodynamic, 55.7% cognitive behavior therapy) reported about 322 outpatients. Therapists were interviewed in person by two research psychotherapists in regard to illness characteristics of unselected patients.

**Results:**

The duration of illness was longer than 1 year in 98.1% of patients or longer than a decade in 54.5%. In the judgement of the therapists 79% of disorders had a chronic or recurrent course. In 25% there were relevant participation impairments in regard to daily activities, leisure time, social relations, or work. About one quarter had already been in inpatient treatment.

**Conclusions:**

The data show that chronic disorders are the rule rather than the exception in psychotherapy. This requires a multidimensional and interdisciplinary treatment approach, including sociomedical interventions in order to sustain participation in life. This should be recognized in the treatment concepts and also get proper attention in the education and reimbursement of psychotherapists.

**Disclosure:**

No significant relationships.

